# Prediction of HELLP Syndrome Severity Using Machine Learning Algorithms—Results from a Retrospective Study

**DOI:** 10.3390/diagnostics13020287

**Published:** 2023-01-12

**Authors:** Marian Melinte-Popescu, Ingrid-Andrada Vasilache, Demetra Socolov, Alina-Sînziana Melinte-Popescu

**Affiliations:** 1 Department of Internal Medicine, Faculty of Medicine and Biological Sciences, ‘Ștefan cel Mare’ University, 720229 Suceava, Romania; 2 Department of Obstetrics and Gynecology, ‘Grigore T. Popa’ University of Medicine and Pharmacy, 700115 Iasi, Romania; 3 Department of Mother and Newborn Care, Faculty of Medicine and Biological Sciences, ‘Ștefan cel Mare’ University, 720229 Suceava, Romania

**Keywords:** HELLP syndrome, severity, prediction, machine learning

## Abstract

(1) Background: HELLP (hemolysis, elevated liver enzymes, and low platelets) syndrome is a rare and life-threatening complication of preeclampsia. The aim of this study was to evaluate and compare the predictive performances of four machine learning-based models for the prediction of HELLP syndrome, and its subtypes according to the Mississippi classification; (2) Methods: This retrospective case-control study evaluated pregnancies that occurred in women who attended a tertiary maternity hospital in Romania between January 2007 and December 2021. The patients’ clinical and paraclinical characteristics were included in four machine learning-based models: decision tree (DT), naïve Bayes (NB), k-nearest neighbors (KNN), and random forest (RF), and their predictive performance were assessed; (3) Results: Our results showed that HELLP syndrome was best predicted by RF (accuracy: 89.4%) and NB (accuracy: 86.9%) models, while DT (accuracy: 91%) and KNN (accuracy: 87.1%) models had the highest performance when used to predict class 1 HELLP syndrome. The predictive performance of these models was modest for class 2 and 3 of HELLP syndrome, with accuracies ranging from 65.2% and 83.8%; (4) Conclusions: The machine learning-based models could be useful tools for predicting HELLP syndrome, and its most severe form—class 1.

## 1. Introduction

HELLP (hemolysis, elevated liver enzymes, and low platelets) syndrome is a rare complication of preeclampsia (PE), associated with high feto-maternal mortality and morbidity rates [1]. The prevalence of HELLP syndrome ranges from 0.5% to 0.9% [2]. The majority of cases (70%) occur during the third trimester of pregnancy or within 48 h after birth, but few of them can manifest as early as the second trimester of pregnancy, after 20 weeks of gestation [3]. The perinatal death rate is approximately 37%, while the maternal mortality rate ranges between 0–24% [4,5].

Maternal complications due to HELLP syndrome are represented by postpartum hemorrhage, placental abruption, heart failure, pulmonary edema, acute kidney injury, disseminated intravascular coagulation (DIC), acute respiratory distress syndrome (ARDS), stroke, and hepatic injury, while neonatal complications include preterm birth, low birth weight, birth asphyxia, neonatal intensive care unit (NICU) admission, and neonatal resuscitation [6,7]. These complications depend on the severity of HELLP syndrome.

The Mississippi triple-class system divides the severity of HELLP syndrome considering the platelet count (PLT), serum asparate aminotransferase (AST) or alanine aminotransferase (ALT) levels, and lactate dehydrogenase (LDH) levels [4]. Class 1 HELLP syndrome is the most severe form, defined as PLT ≤ 50.000/mm^3^, AST or ALT ≥ 70 IU/L, and serum LDH ≥ 600 IU/L. Class 2 is the moderate form of HELLP syndrome, characterized by a platelet number between 50.000–100.00/mm^3^, AST or ALT ≥ 70 IU/L, and serum LDH ≥ 600 IU/L. Finally, class 3 is the least severe form of HELLP syndrome, diagnosed based on a platelet number between 100.000–150.00/mm^3^, AST or ALT ≥ 40 IU/L, and serum LDH ≥ 600 IU/L.

The physiopathology of this disorder is mainly based on the ischemic placental tissue that leads to endothelium activation, increased synthesis of antiangiogenic factors, and microvascular injury [8]. Other components of this syndrome include abnormal oxidation of fatty acids by the fetus, inflammation, and coagulation cascade activation, leading to thrombocytopenia, microangiopathic hemolytic anemia, multiorgan microvascular injury and hepatic necrosis [8]. Moreover, a recent bioinformatics analysis demonstrated a genetic component for HELLP syndrome and proposed multiple therapeutic targets for further studies [9].

Considering the multiple life-threatening complications of this syndrome, it is vital to offer an individualized management, and to try to identify potential predictors for disease severity and progression. In a cross-sectional retrospective study, the authors discovered a superior predictive performance of aspartate-aminotransferase to platelet ratio index (APRI) score compared to AST serum levels in terms of sensitivity (82.6% versus 71.7%), but not specificity (87.6% versus 91.2%) for HELLP syndrome prediction [10]. Oliveira et al. evaluated the first-trimester maternal characteristics and biomarkers in pregnancies that subsequently develop HELLP syndrome, and identified as main risk factors for this disorder the following: Caucasian ethnicity, nulliparity, history of previous PE, history of previous HELLP syndrome, and first-trimester mean arterial pressure (MAP) [11]. Moreover, Lind Malte et al. assessed the performance of a combination of angiogenic and vasoactive biomarkers to predict the development of severe preeclampsia/HELLP syndrome in the third trimester of pregnancy, and determined that a combination of biomarkers (CT-pro-ET-1, sFlt-1, and systolic blood pressure) achieved an AUC of 0.94 for prediction of development of severe PE/HELLP syndrome within 1 week, and an AUC of 0.83 for prediction of their development within 2 weeks of evaluation [12].

On the other hand, since HELLP syndrome is a complication of PE, conventional risk factors and biomarkers used for PE prediction could be used to assess the risk of developing this disorder. An unicentric cross-sectional study of high-risk pregnancies that compared the levels of soluble fms-like tyrosine kinase-1 (sFlt-1)/placental growth factor (PLGF) ratio and soluble endoglin (sEng) for the detection of placental-related disorders identified an area under the curve (AUC) value of 0.895 (95%-Cl 0.83–0.96) for sFlt-1/PLGF ratio and 0.878 (95%-Cl 0.81–0.95) for sEng when considering HELLP syndrome [13].

The new trend in predictive medicine is the use of various machine learning techniques and artificial neural networks for the prediction of a disease. In a recent study, the authors developed a neuro-fuzzy model for HELLP syndrome prediction in mobile cloud computing environments using the pregnant patient’s symptoms, and obtained good predictive performance (precision: 0.685, recall: 0.756, the F-score: 0.705, and AUC: 0.829) [14]. Further, for preeclampsia prediction, various machine learning approaches were developed such as random forest (RF), decision trees (DT), gradient boosting (GB), naïve Bayes (NB), and support vector machine (SVM), with good predictive performance [15,16,17,18].

The aim of this study was to evaluate and compare the predictive performances of four machine learning-based models for the prediction of HELLP syndrome and its subtypes according to the Mississippi classification.

## 2. Materials and Methods

### 2.1. Study Design and Informed Consent

We conducted a retrospective case-control study of pregnancies that occurred in women who attended a tertiary maternity hospital: ‘Cuza-Voda’, Iasi, Romania, between January 2007 and December 2021. Ethical approval for this study was obtained from the Institutional Ethics Committee of University of Medicine and Pharmacy ‘Grigore T. Popa’ (No. 151/13.02.2022). Informed consent was obtained from all participants included in the study. All methods were carried out in accordance with relevant guidelines and regulations.

### 2.2. Study Population

We recruited participants at the time of admission to our tertiary center. The inclusion criteria taken into consideration were as follows: pregnant patients with singleton pregnancies who developed HELLP syndrome, maternal age ≥ 18, and certain first trimester pregnancy dating. Exclusion criteria comprised patients who had multiple pregnancies, fetuses with chromosomal or structural abnormalities, intrauterine infection, incomplete medical records, incorrect/lack of first trimester sonographic pregnancy dating, or who were unable to offer informed consent.

### 2.3. Data Collection

Maternal characteristics and previous medical history were evaluated by a physician, and maternal risk factors for preeclampsia and HELLP syndrome were recorded in the database. The following parameters were evaluated: demographic data, age, parity, BMI (body mass index), smoking status during pregnancy, the use of ART, obstetrical comorbidities, personal history of PE, diabetes, pre-existing chronic hypertension, renal disease, hepatic disease, thrombophilia, systemic lupus erythematosus (SLE), antiphospholipid syndrome (APS), serum values of LDH, AST, PLT, highest systolic (SBP), and diastolic (DBP) blood pressure values, symptoms, HELLP syndrome complications (eclampsia, abruptio placentae, pulmonary edema, hepato-renal syndrome, hepatic insufficiency, sepsis), and number of maternal deaths.

The APRI was calculated using the following formula: [AST (IU/l)/AST (Upper Limit of Normal-IU/l)/Platelet count (10^9^/l)] × 100. Blood pressure was measured using the Fetal Medicine Foundation (FMF) guidelines [19] with a calibrated device (Omron M3 COMFORT; Omron Corp, Kyoto, Japan). The following pregnancy outcomes were recorded: type of birth, presentation, gestational age at birth, newborn’s gender, birthweight, length, Apgar scores at 1 and 5 min, NICU admission, neonatal death.

### 2.4. Study Groups

A total of 161 patients were included in the analysis of this study and were divided into two groups: those who developed HELLP syndrome (81 patients, group 1), and those who did not develop HELLP syndrome (80 patients, group 2). The pregnant patients affected by HELLP syndrome were subsequently divided into the following subgroups according to the Mississippi classification: subgroup 1 (Class 1, *n* = 21), subgroup 2 (Class 2, *n* = 35), and subgroup 3 (Class 3, *n* = 25).

### 2.5. First Step: Statistical Analysis

In the first stage of the statistical analysis, each variable was evaluated with chi-squared and Fisher’s exact tests for categorical variables, which were presented as frequencies with corresponding percentages, and *t*-tests for continuous variables, which were presented as means and standard deviations (SD).

ANOVA analysis with the Bonferroni post-hoc test was used to determine whether there is a statistically significant difference between the subgroups regarding their paraclinical characteristics (AST, LDH, PLT, APRI, SBP, DBP), and boxplots were used for graphical representations of these differences. The statistical analyses were performed using STATA SE (version 14, 2015, StataCorp LLC, College Station, TX, USA).

### 2.6. Machine Learning Analysis- Data Inclusion and Types of Algorithms

In the second stage of the analysis, we evaluated the predictive performance of four machine learning-based models: decision tree, naïve Bayes, k-nearest neighbors (KNN), and random forest algorithm. Clinical data included in the evaluation were as follows: demographic, age, parity, BMI, smoking status during pregnancy, the use of ART, obstetrical comorbidities, and pathologic personal history (PE, diabetes, pre-existing chronic hypertension, renal disease, hepatic disease, thrombophilia, SLE, APS). The paraclinical data included: APRI score, SBP, and DBP.

### 2.7. Feature Selection and Data Processing

Our first step in helping the data mining process was to standardize the parameters using AutoData Prep from the field operations. No feature selection was employed because we wanted to assess the overall predictive performance of a combination of clinical and paraclinical parameters that are routinely determined by clinicians.

Data were further segregated into data for testing (70%) and training (30%). In order to protect from overfitting, all models underwent 5-fold cross validation. After cross validation of both testing and training data, we calculated the models’ predictive performance based on the training results. Their true positive rates (TPR), false negative rates (FNR), false detection rates (FDR), accuracies, values for area under the receiver operating characteristic curve (AUROC), precision, and F1 scores were calculated, and compared for HELLP syndrome, class 1, 2, and 3 subgroups, respectively. The comparison was made using ROC analysis, and the results were plotted. The models were constructed and analyzed using IBM SPSS Modeler (version 1.0.0.399, IBM Corporation, Armonk, NY, USA).

## 3. Results

### 3.1. Clinical and Paraclinical Characteristics of the Patients Included in the Main Groups

A total of 161 pregnant patients were evaluated in our retrospective study. Their clinical and paraclinical characteristics are presented in Table 1, segregated into the following groups: those who developed HELLP syndrome (81 patients, group 1), and those who did not develop HELLP syndrome (80 patients, group 2).

The first group had significantly more patients with a personal history of chronic hypertension (*p* = 0.024), SLE/APS (*p* = 0.004), thrombophilia (*p* = 0.007), and preeclampsia in previous pregnancies (*p* = 0.004). Moreover, obesity (*p* < 0.001), nulliparity (*p* = 0.021), and the use of ART (*p* = 0.007) were significantly more frequently encountered in the first group compared to the second group (*p* < 0.001). Regarding the evaluated paraclinical characteristics, the APRI score, SBP, and DBP were significantly higher for the HELLP group (*p* < 0.001).

### 3.2. Pregnancy Outcomes of the Patients Included in the Main Groups

Pregnancy outcomes for the main groups are presented in Table 2. Pregnancies affected by HELLP syndrome were significantly associated with complications such as preterm birth (*p* < 0.001), intrauterine growth restriction (*p* < 0.001), and oligoamnios (*p* = 0.01). The patients in the first group had a significantly higher cesarean delivery rate (*n* = 77 patients, 95.06%; *p* < 0.001) and their newborns had significantly lower birthweight, Apgar scores at 1 and 5 min, and length (*p* < 0.001). Moreover, significantly more newborns from mothers with HELLP syndrome were admitted to NICU for specific treatment (*p* = 0.015), and three newborn deaths were recorded. Overall, three maternal deaths (3.7%) were recorded in the HELLP syndrome group, while only one neonatal death was recorded in the control group. Hepatic insufficiency, pulmonary edema, and sepsis (*n* = 1, 1.25%), and hepatorenal syndrome (*n* = 3, 3.7%) were among the complications recorded in the main group.

### 3.3. Subgroups Comparisons

**Figure 1:**
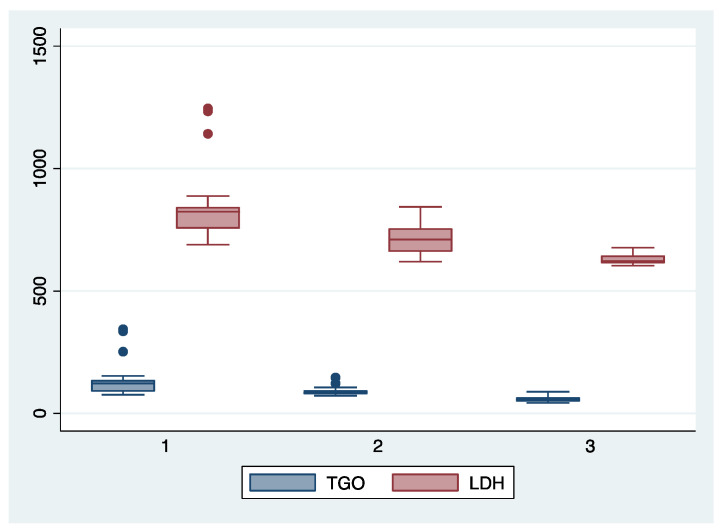
Boxplot representing the comparison of AST (TGO) and LDH serum valued in the subgroups 1, 2, and 3. Legend: LDH—lactate dehydrogenase; TGO—asparate aminotransferase.

**Figure 2:**
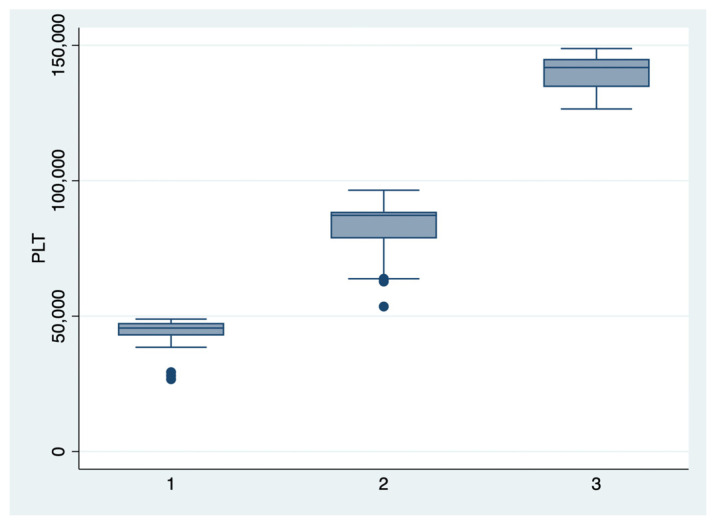
Boxplot representing the comparison of the platelets number in the subgroups 1, 2, and 3. Legend: PLT—platelets.

**Figure 3:**
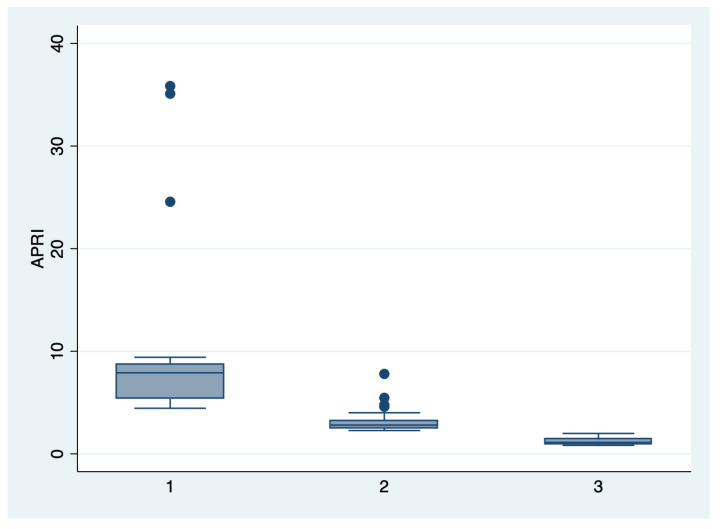
Boxplot representing the comparison of the APRI score in the subgroups 1, 2, and 3. Legend: APRI—aspartate-aminotransferase to platelet ratio index.

**Figure 4:**
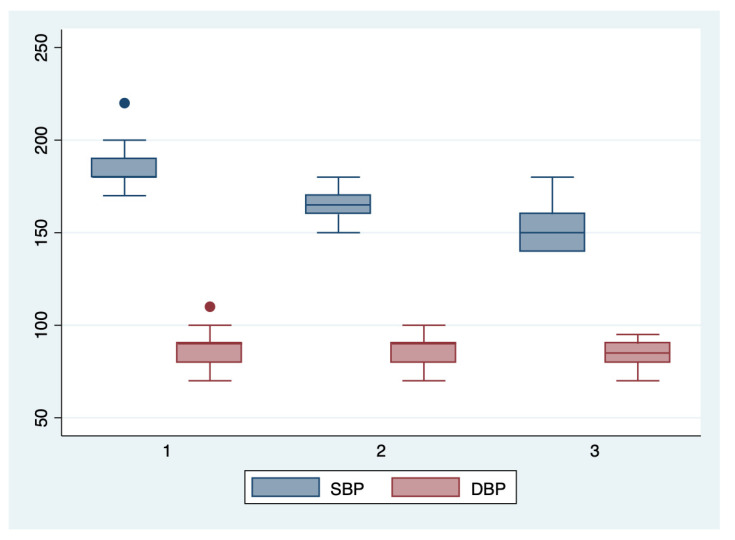
Boxplot representing the comparison of the systolic and diastolic blood pressures in the subgroups 1, 2, and 3. Legend: SBP—systolic blood pressure; DBP—diastolic blood pressure.

We further comparatively analyzed the paraclinical characteristics and symptoms of the following subgroups: subgroup 1 (Class 1, *n* = 21), subgroup 2 (Class 2, *n* = 35), and subgroup 3 (Class 3, *n* = 25) (Table 3 and Table 4). The serum values of LDH and AST were significantly higher, and the number of platelets was significantly lower for the first subgroup when compared to the second and third subgroups (*p* < 0.001). Moreover, we observed an ascending trend for APRI, SBP, and DBP values from the third subgroup to the first one, although the latter did not reach a statistically significant level (*p* = 0.21). Graphical representations of the subgroup’s comparisons are represented in Figure 1, Figure 2, Figure 3 and Figure 4. Although all the analyzed symptoms were more prevalent in the second subgroup, we could not find any statistically significant differences between subgroups.

### 3.4. The Predictive Performance of Machine Learning-Based Models for the HELLP Syndrome and Its Subtypes

In the second stage of the analysis, we incorporated the pregnant patient’s clinical and paraclinical characteristics into four machine learning-based models, and we calculated their predictive performance (Table 5). DT achieved the highest accuracy when predicting class 1 HELLP syndrome (91%), with an TPR value of 94.9%. Although the RF algorithm had the highest TPR value for class 1 HELLP prediction (88.6%), its best performance was achieved when used to predict all types of HELLP syndrome, with an accuracy of 89.4%. A similar situation described the predictive performance of NB model, which achieved an accuracy of 86.9% for all types of HELLP syndrome, and a TPR of 88.3% for class 3 HELLP syndrome. Finally, the KNN model appeared to have the highest predictive performance when used to predict class 1 HELLP syndrome, achieving an accuracy of 87.1%, and an AUROC value of 0.81.

### 3.5. ROC Analysis

**Figure 5:**
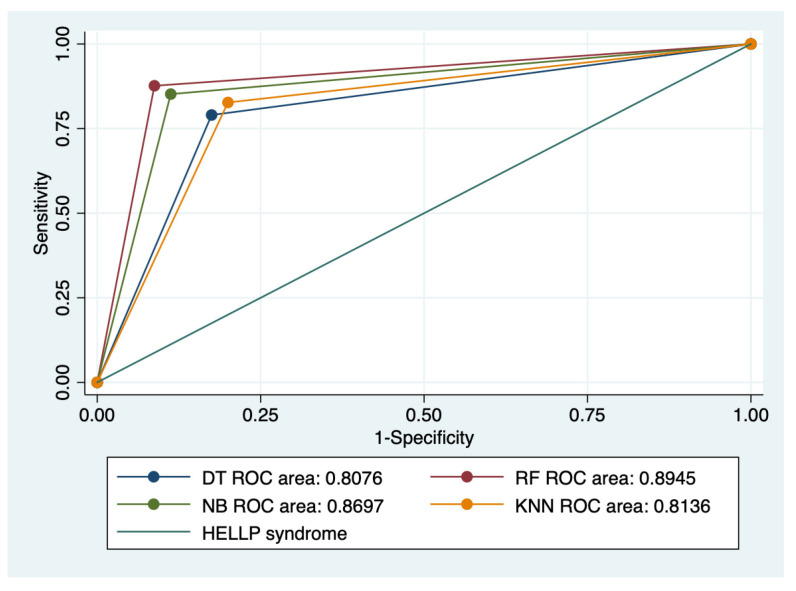
Multiple ROC curves comparison between different machine learning models for all types of HELLP syndrome. Legend: ROC—Receiver operating characteristic; DT—decision trees; NB—naïve Bayes; KNN—k-nearest neighbors; RF—random forest.

**Figure 6:**
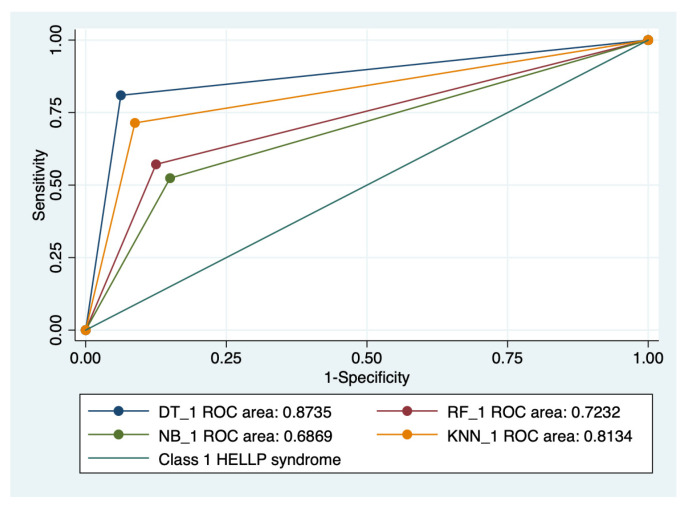
Multiple ROC curves comparison between different machine learning models for class 1 HELLP syndrome. Legend: ROC—Receiver operating characteristic; DT—decision trees; NB—naïve Bayes; KNN—k-nearest neighbors; RF—random forest.

**Figure 7:**
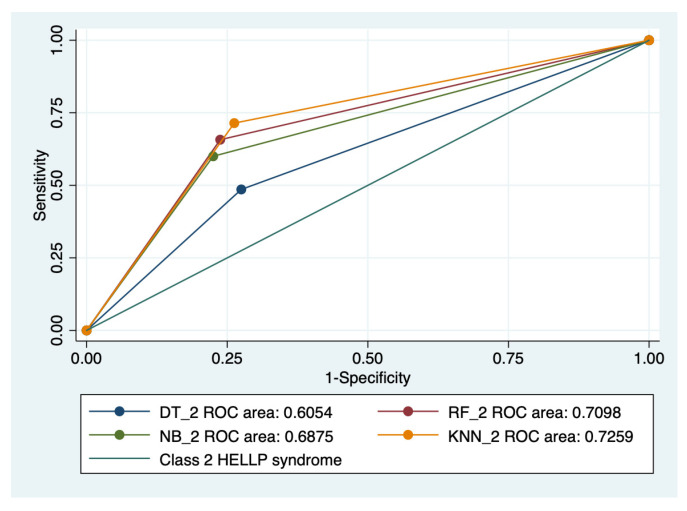
Multiple ROC curves comparison between different machine learning models for class 2 HELLP syndrome. Legend: ROC—Receiver operating characteristic; DT—decision trees; NB—naïve Bayes; KNN—k-nearest neighbors; RF—random forest.

**Figure 8:**
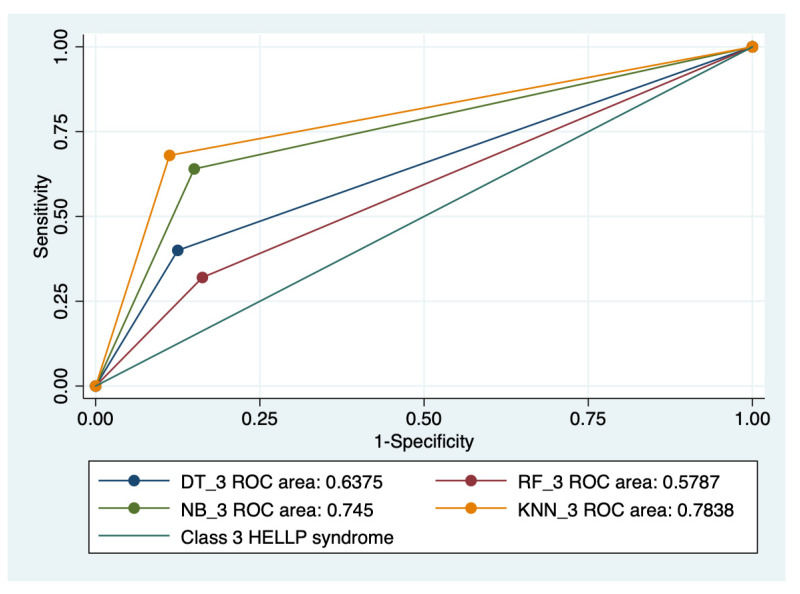
Multiple ROC curves comparison between different machine learning models for class 3 HELLP syndrome. Legend: ROC—Receiver operating characteristic; DT—decision trees; NB—naïve Bayes; KNN—k-nearest neighbors; RF—random forest.

Graphical representations of multiple ROC curves comparisons between machine learning algorithms in relationship with the evaluated groups are presented in Figure 5, Figure 6, Figure 7 and Figure 8, and the results from the ROC analysis are indicated in Table 6. The latter analysis confirmed a statistically significant difference (*p* < 0.05) regarding the AUROC values of various ML models in relationship with the evaluated groups.

## 4. Discussion

This is the first retrospective study in the literature that trained four machine-learning based models (DT, NB, KNN, and RF) for the prediction of HELLP syndrome and its severity (three classes) in a cohort of pregnant patients with singleton pregnancies, using clinical and paraclinical characteristics.

Our results showed that HELLP syndrome was best predicted by RF (accuracy: 89.4%; AUROC: 0.89), and NB (accuracy: 86.9%; AUROC: 0.86) models, while DT (accuracy: 91%; AUROC: 0.87), and KNN (accuracy: 87.1%; AUROC: 0.81) models had the highest performance when used to predict class 1 HELLP syndrome. The predictive performance of these models was modest for class 2 and 3 of HELLP syndrome, with accuracies ranging from 65.2% and 83.8%.

The bagging algorithm serves as the foundation for RF, which employs ensemble learning [20]. It creates as many trees on the subset of the data and combines the output of all the trees. In doing so, it lessens the issue of overfitting in decision trees, as well as lowering variance and raising accuracy. On the other hand, NB is suitable for solving multi-class prediction problems, especially when using small datasets, and has much lower costs than RF [21]. DT models are similar to NB in terms of handling small datasets and low costs, but have the disadvantage of overfitting without a proper data standardization process [22]. Finally, one of the biggest advantages of the KNN model is that it can be used both for classification and regression problems, but does not perform well on imbalanced data [23].

In a recent review by Uddin et al., the authors aimed to investigate the predictive performance of various machine learning approaches and showed that for papers that used only clinical and demographic data, DT had the highest accuracy, while for those articles that used research data other than ‘clinical and demographic’ type, SVM and RF have been found to show the superior accuracy at most times [24]. Another retrospective study that evaluated the predictive performance of two ML models for the prediction of adverse outcomes, including HELLP syndrome, in patients with suspected preeclampsia, demonstrated similar performances in terms of positive predictive values for gradient-boosted tree model (88 ± 6%) and random forest classifier (88 ± 6%) [16].

Our models used both clinical and paraclinical data, and showed superior predictive performance for the severe form of HELLP syndrome (class 1), which constitutes an advantage for physicians who follow the clinical progression of this disorder. The clinical characteristics can be easily obtained from the patient’s anamnesis and medical records, while the paraclinical characteristics used, including APRI score, could be rapidly determined in the local hospitals, allowing a possible anticipation of the pregnancy’s adverse outcome.

Pregnancies affected by HELLP syndrome presented significantly more severe outcomes such preterm birth (*n* = 60, 74.07%), intrauterine growth restriction (*n* = 33, 40.74%), oligoamnios (*n* = 13, 16.05%), low Apgar scores at 1 (6.44 ± 2.31) and 5 (6.67 ± 2.65) minutes of the newborns, and more NICU admissions (*n* = 13, 16.05%). A recent retrospective study by Li et al. that analyzed the similarities and differences in the clinical features and pregnancy outcomes in various forms of PE and HELLP syndrome reported similar results, and a global incidence of adverse maternal outcomes of 61.4% [25].

Our study has several limitations, including a small cohort of patients and number of predictors, but at the same time, the trained models have the advantage of an easier implementation by the physicians. All chosen machine learning-based models have the ability to handle small sample data [26]. Moreover, the used algorithms have proven superior predictive performance when applied for datasets based mainly on categorical predictors in comparison with other models such as gradient boosting [27], artificial neural networks [28], support vector machines, extreme gradient boosting, multilayer perceptron [29], or linear discriminant analysis [30].

We hypothesize that the model’s accuracy could be improved by adding specific sonographic and serum markers for preeclampsia prediction, since their physiopathology is closely related [31,32,33,34,35]. On the other hand, this is the first study in the literature that evaluated the predictive performance of four machine learning-based models for the prediction of HELLP syndrome and its subtypes on a cohort of pregnant patients from a tertiary center during a 14-year timeframe.

Further studies on larger cohorts of patients could evaluate the predictive performance of these ML-based models in different settings and populations. The results could aid clinicians in the risk stratification process of pregnant patients and could help calculate the risk–benefit ratio in order to support the decision of prompt delivery versus conservative management of the case.

## 5. Conclusions

This is the first retrospective study in the literature that trained four machine learning-based models based on clinical and paraclinical characteristics for the prediction of HELLP syndrome and its severity in a cohort of pregnant patients with singleton pregnancies.

Pregnancies affected by HELLP syndrome were associated with significantly more adverse pregnancy outcomes such as preterm birth, intrauterine growth restriction, oligoamnios, low Apgar scores at 1 and 5 min, and more NICU admissions.

The results from our study indicated that HELLP syndrome was best predicted by RF and NB models, while DT and KNN models had the highest performance when used to predict class 1 HELLP syndrome.

Further studies on larger cohorts of patients could demonstrate an improvement of the predictive performance of these models by adding traditional markers for preeclampsia.

## Figures and Tables

**Table 1:** Clinical and paraclinical characteristics of the patients included in the main groups.

Patient’s Characteristics	Group 1 (HELLP, *n* = 81)	Group 2 (without HELLP, *n* = 80)	*p* Value
Age, years (mean ± SD)	29.92 ± 6.92	28.48 ± 6.76	0.18
Medium (*n*/%)	Urban = 33 (40.7%) Rural = 48 (59.3%)	Urban = 33 (41.2%) Rural = 47 (58.8%)	0.94
Parity (*n*/%)	Nulliparity = 54 (66.7%) Multiparity = 27 (33.3%)	Nulliparity = 39 (48.8%) Multiparity = 41 (51.2%)	0.021
Obesity (*n*/%)	Yes = 21 (25.9%)	Yes = 2 (2.5%)	<0.001
Use of ART (*n*/%)	Yes = 7 (8.6%)	Yes = 0 (0%)	0.007
Smoking (*n*/%)	Yes = 0 (0%)	Yes = 2 (2.5%)	0.15
Placenta praevia (*n*/%)	Yes = 1 (1.2%)	Yes = 9 (11.3%)	0.008
Personal history of adverse pregnancy outcomes (*n*/%)	Yes = 10 (12.3%)	Yes = 4 (5%)	0.098
Personal history of PE (*n*/%)	Yes = 8 (9.9%)	Yes = 0 (0%)	0.004
Personal history of chronic hypertension (*n*/%)	Yes = 5 (6.2%)	Yes = 0 (0%)	0.024
Personal history of renal disease (*n*/%)	Yes = 1 (1.2%)	Yes = 0 (0%)	0.31
Personal history of diabetes (*n*/%)	Yes = 1 (1.2%)	Yes = 0 (0%)	0.31
Personal history of hepatic disease (*n*/%)	Yes = 1 (1.2%)	Yes = 0 (0%)	0.31
Personal history of SLE/APS (*n*/%)	Yes = 8 (9.9%)	Yes = 0 (0%)	0.004
Personal history of thrombophilia (*n*/%)	Yes = 7 (8.6%)	Yes = 0 (0%)	0.007
APRI score (mean ± SD)	4.49 ± 5.99	0.31 ± 0.05	<0.001
SBP, mmHg (mean ± SD)	167.01 ± 16.59	117.5 ± 10.82	<0.001
DBP, mmHg (mean ± SD)	85.30 ± 7.71	75.12 ± 5.45	<0.001

Table legend: ART—assisted reproduction techniques; PE—preeclampsia; SD—standard deviation; APS—antiphospholipid syndrome; SLE—systemic lupus erythematosus; APRI—aspartate-aminotransferase to platelet ratio index; SBP—systolic blood pressure; DBP—diastolic blood pressure.

**Table 2:** Pregnancy outcomes of the patients included in the main groups.

Pregnancy Outcome	Group 1 (HELLP, *n* = 81)	Group 2 (without HELLP, *n* = 80)	*p* Value
Preterm birth (*n*/%)	Yes = 60 (74.07%)	Yes = 15 (18.75%)	<0.001
Intrauterine growth restriction (*n*/%)	Yes = 33 (40.74%)	Yes = 5 (6.25%)	<0.001
Oligoamnios (*n*/%)	Yes = 13 (16.05%)	Yes = 3 (3.75%)	0.015
Polyhydramnios (*n*/%)	Yes = 0 (0%)	Yes = 2 (2.5%)	0.02
PE related complications (*n*/%)	Eclampsia = 3 (3.70%) Abruptio placentae = 1 (1.23%)	-	-
Pulmonary edema (*n*/%)	Yes = 1 (1.25%)	Yes = 0 (0%)	0.29
Hepatic insufficiency (*n*/%)	Yes = 1 (1.25%)	Yes = 0 (0%)	0.29
Sepsis (*n*/%)	Yes = 1 (1.25%)	Yes = 0 (0%)	0.29
Hepatorenal syndrome (*n*/%)	Yes = 3 (3.7%)	Yes = 0 (0%)	0.08
Newborn’s gender (*n*/%)	Male = 47 (58.02%) Female = 34 (41.98%)	Male = 41 (51.25%) Female = 39 (48.75%)	0.38
Gestational age at birth, weeks (mean ± SD)	34.14 ± 3.24	37.52 ± 2.70	<0.001
Mode of delivery (*n*/%)	Cesarean = 77 (95.06%) Vaginal = 4 (4.94%)	Cesarean = 46 (57.50%) Vaginal = 34 (42.50%)	<0.001
Presentation (*n*/%)	Cephalic = 67 (82.72%) Breech = 11 (13.58%) Transverse = 3 (3.7%)	Cephalic = 75 (93.75%) Breech = 4 (5.00%) Transverse = 1 (1.25%)	0.10
Apgar score at 1 min (mean ± SD)	6.44 ± 2.31	8.12 ± 1.15	<0.001
Apgar score at 5 min (mean ± SD)	6.67 ± 2.65	8.6 ± 0.86	<0.001
Birthweight, g (mean ± SD)	2038.02 ± 728.64	3075.37 ± 724.43	<0.001
Newborn’s length, cm (mean ± SD)	44.2 ± 6.75	49.68 ± 3.85	<0.001
NICU admission (*n*/%)	Yes = 13 (16.05%)	Yes = 3 (3.75%)	0.015
Neonatal death (*n*/%)	Yes = 3 (3.7%)	Yes = 1 (1.25%)	0.31
Maternal death (*n*/%)	Yes = 3 (3.7%)	Yes = 0 (0%)	0.08

Table legend: PE—preeclampsia; SD—standard deviation; g- grams; NICU—neonatal intensive care unit.

**Table 3:** Comparison of paraclinical characteristics for the patients included in the analyzed subgroups.

Paraclinical Parameter	Subgroup 1 (Class 1, *n*= 21)	Subgroup 2 (Class 2, *n*= 35)	Subgroup 3 (Class 3, *n*= 25)	Sum of Squares between Groups	F Score	*p* Value
LDH, IU/L (mean ± SD)	851.66 ± 158.53	708.45 ± 55.47	631.32 ± 23.09	565,069.208	35.54	<0.001
AST, IU/L (mean ± SD)	140.8 ± 76.24	87.77 ± 14.63	59.36 ± 13.40	280,439.83	114.14	<0.001
PLT, number/mm^3^ (mean ± SD)	42,966.66 ± 6807.07	83,312 ± 9785.08	139,570 ± 6886.97	6.21	428.69	<0.001
APRI score (mean ± SD)	10.66 ± 9.22	3.12 ± 1.08	1.22 ± 0.33	1836.47	55.10	<0.001
SBP, mmHg (mean ± SD)	185.61 ± 11.44	167 ± 8.59	151.4 ± 12.12	112,031.99	327.42	<0.001
DBP, mmHg (mean ± SD)	86.66 ± 8.85	85.42 ± 7.41	84 ± 7.21	4256.07	31.67	0.21

Table legend: LDH—lactate dehydrogenase; AST—asparate aminotransferase; PLT—platelets; SD—standard deviation; APRI—aspartate-aminotransferase to platelet ratio index; SBP—systolic blood pressure; DBP—diastolic blood pressure.

**Table 4:** Comparison of the main symptoms between analyzed subgroups.

Symptoms	Subgroup 1 (Class 1, *n* = 21)	Subgroup 2 (Class 2, *n* = 35)	Subgroup 3 (Class 3, *n* = 25)	*p* Value
Headache (*n*/%)	Yes = 11 (13.6%)	Yes = 16 (19.8%)	Yes = 8 (9.9%)	0.35
Nausea (*n*/%)	Yes = 11 (13.6%)	Yes = 17 (21%)	Yes = 10 (12.3%)	0.68
Edema (*n*/%)	Yes = 14 (17.3%)	Yes = 20 (24.7%)	Yes = 14 (17.3%)	0.72
Visual disturbances (*n*/%)	Yes = 7 (8.6%)	Yes = 12 (14.8%)	Yes = 4 (4.9%)	0.25
Epigastric pain (*n*/%)	Yes = 15 (18.5%)	Yes = 23 (28.4%)	Yes = 8 (9.9%)	0.35

**Table 5:** The predictive performance of machine learning-based models for the HELLP syndrome and its subtypes.

ML Model	Type of HELLP	TPR (%)	FNR (%)	FDR (%)	Accuracy (%)	AUROC Value	Precision	F1 Score
DT	All HELLP	79.5	20.4	17.5	80.7	0.80	0.82	0.80
	Class 1	94.9	5	6	91	0.87	0.93	0.94
	Class 2	76.3	23.6	27.5	65.2	0.60	0.72	0.74
	Class 3	82.3	17.6	12.5	76.1	0.63	0.87	0.84
RF	All HELLP	87.9	12	8	89.4	0.89	0.91	0.89
	Class 1	88.6	11.3	12.5	81.1	0.72	0.87	0.88
	Class 2	83.5	16.4	23.7	73	0.70	0.76	0.79
	Class 3	79.7	20.2	16.2	71.4	0.57	0.83	0.81
NB	All HELLP	85.5	14.4	11.2	86.9	0.86	0.88	0.87
	Class 1	87.1	12.8	15	78.2	0.68	0.85	0.86
	Class 2	81.5	18.4	22.5	72.1	0.68	0.77	0.79
	Class 3	88.3	11.6	15	80	0.74	0.85	0.86
KNN	All HELLP	82	17.9	20	81.3	0.81	0.80	0.81
	Class 1	92.4	7	8	87.1	0.81	0.91	0.91
	Class 2	85.5	14.4	26.2	73	0.72	0.73	0.79
	Class 3	89.8	10.1	11.2	83.8	0.78	0.88	0.89

Table legend: All HELLP—all types of HELLP syndrome; ML—machine learning; DT—decision trees; NB—naïve Bayes; KNN—k-nearest neighbors; RF—random forest; TPR—true positive rate; FNR—false negative rate; FDR—false detection rate; AUROC—area under the receiver operating characteristic curve.

**Table 6:** The results from ROC analysis of machine learning-based models for the HELLP syndrome and its subtypes.

Type of HELLP	ML Model	AUROC Value	Standard Error	95% CI Lower Limit	95% CI Upper Limit	*p* Value
All HELLP	DT	0.80	0.03	0.74	0.86	0.001
	NB	0.86	0.02	0.81	0.92	
	RF	0.89	0.02	0.84	0.94	
	KNN	0.81	0.03	0.75	0.87	
Class 1	DT	0.87	0.04	0.78	0.96	0.006
	NB	0.68	0.05	0.57	0.80	
	RF	0.72	0.05	0.60	0.83	
	KNN	0.81	0.05	0.70	0.91	
Class 2	DT	0.60	0.04	0.50	0.70	0.008
	NB	0.68	0.04	0.59	0.78	
	RF	0.70	0.04	0.61	0.80	
	KNN	0.72	0.04	0.63 1	0.81	
Class 3	DT	0.63	0.05	0.53	0.74	<0.001
	NB	0.74	0.05	0.64	0.84	
	RF	0.57	0.05	0.47	0.68	
	KNN	0.78	0.05	0.68	0.88	

Table legend: All HELLP—all types of HELLP syndrome; ML—machine learning; DT—decision trees; NB—naïve Bayes; KNN—k-nearest neighbors; RF—random forest; AUROC—area under the receiver operating characteristic curve; CI—confidence interval.

## Data Availability

The data presented in this study are available on request from the corresponding author. The data are not publicly available due to local policies.
